# Monitoring of CRT-D devices during radiation therapy in vitro

**DOI:** 10.1186/s12938-016-0144-7

**Published:** 2016-03-09

**Authors:** Martin Augustynek, David Korpas, Marek Penhaker, Jakub Cvek, Andrea Binarova

**Affiliations:** Institute of Nursing, Faculty of Public Policies, Silesian University, Opava, Czech Republic; Department of Cybernetics and Biomedical Engineering, Faculty of Electrical Engineering and Computer Science, VSB – Technical University, Ostrava, Czech Republic; Oncology clinic, University Hospital Ostrava, Ostrava, Czech Republic

**Keywords:** Implantable cardioverter defibrillator, Radiation, Interference, Cardiac resynchronization therapy

## Abstract

**Background:**

Using of active cardiac medical devices increases steadily. In Europe, there were 183 implants of ICD and 944 implants of PM, 119 of biventricular ICD and 41 of biventricular PM, all per million inhabitants in 2014. Healthcare environments, including radiotherapy treatment rooms, are considered challenging for these implantable devices. Exposure to radiation may cause the device to experience premature elective replacement indicator, decreased pacing amplitude or pacing inhibition, inappropriate shocks or inhibition of tachyarrhythmia therapy and loss of device function. These impacts may be temporary or permanent. The aim of this study was to evaluate the influence of linear accelerator ionizing radiation dose of 10 Gy on the activity of the biventricular cardioverter-defibrillator in different position in radiation beam.

**Methods:**

Two identical wireless communication devices with all three leads were used for the measurement. Both systems were soused into solution saline and exposed in different position in the beam of linear accelerator per 10 Gy fractions. In comparison of usually used maximum recommended dose of 2 Gy, the radiation doses used in test were five times higher. Using the simultaneous monitoring wireless communication between device and its programmer allowed watching of the devices activities, noise occurrence or drop of biventricular pacing on the programmer screen, observed by local television loop camera.

**Results:**

At any device position in radiation beam, there were no influences of the device activity at dose of 10 Gy neither a significant increase of a solution saline temperature in any of the measured positions of CRT-D systems in linear accelerator.

**Conclusions:**

The results of the study indicated, that the recommendation dose for treating the patients with implantable devices are too conservative and the risk of device failure is not so high. The systems can easily withstand the dose fractions of tens Gy, which would allow current single-dose-procedure treatment in radiation therapy. Even though the process of the random alteration of device memory and electrical components by scatter particles not allowed to specify a safe dose during ionizing radiation, this study showed that the safe limit are above the today used dose fractions.

## Background

Biventricular cardioverter-defibrillators (CRT-D) are life-saving devices that are used in cardiac resynchronization therapy (CRT). CRTs involve the synchronization of both ventricles in order to maintain ventricular contraction towards the septum. This requires a permanent pacing using two leads implanted in the right ventricle and in the coronary veins of the left ventricle. CRTs are currently among the standard procedures used for the non-pharmacological treatment of severe heart failure and have been extensively used in recent years in the therapeutic management of patients with end-stage heart failure [1] to increase the ejection fraction. The evidence of the clinical benefit of CRTs offers randomized studies including patients with QRS > 120 ms as a marker of ventricular dyssynchrony.

The CRT-D implant rate has been steadily increasing worldwide. By conservative estimates, at least one million patients in the USA have permanent artificial cardiac pacemakers [2] and this figure could be in excess of five million patients worldwide [3–5]. These patients may also need to be treated using radiation therapy and any oncology department should expect at least several patients with these devices coming in every year. Radiation therapy is known to have an effect on pacemaker and implantable cardioverter-defibrillator function [6] via electromagnetic interference or through the effects of ionizing radiation [7–11]. The effects of radiation therapy on pacemakers has been studied in detail in the past [11–16]. Based on the studies’ results, the American Association of Physicists in Medicine (AAPM) presented recommendations for irradiation of pacemaker patients in 1994 [7] and divided the hazards into two categories—electromagnetic noise interference and radiation damage. The studies mentioned in the AAPM report encompass older pacemaker types based on an old dipole and the first generation of complementary metal oxide semiconductor (CMOS) technology. Modern pacemakers, using newer CMOS circuitry, differ from these devices both in their sensitivity to radiation and in the type of malfunction observed [10]. The effect of irradiation on CMOS technology has also already been described in detail [8].

**Fig. 1:**
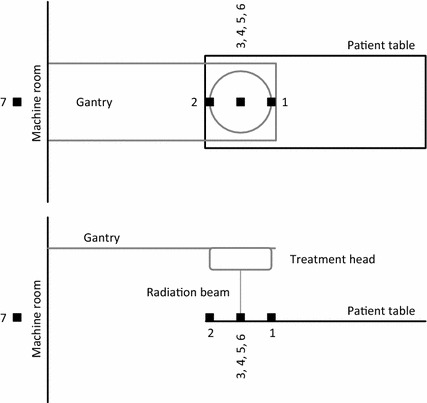
Linear accelerator measurement setup. The numbers represent the cases in the text. Horizontal projection (*above*), side projection (*below*)

In the case of implanted cardiac devices, the workflow of treatment planning and delivery is specific for different departments. It is necessary to minimize the dose for devices with aid of contouring as an “organ” at risk. We usually use treatment plans with a minimal number of monitor units and with the shortest treatment time. Pacemaker functioning is checked before and after the first fraction as well as after the radiation course is administered (Fig. 1).

The novelty of our study lies in the unique simultaneous monitoring of the devices during radiation sessions. In previous studies, the devices were just inspected before and after the radiation session by accessing the system, without the possibility of checking the maintaining of the pacing [17–20].

## Methods

To take measurements, two identical biventricular cardioverter-defibrillators, the Cognis model P107 Boston Scientific (Natick, MA, USA) with wireless communication between the device and programmer, were used. Commonly-used leads were attached to the devices: The Fineline Sterox II model 4459 (right atrium port), the Endotak Reliance SG model 0182 (right ventricle and defibrillation ports) and the Acuity Easyaccess model 4554 (left ventricle port). This system was placed into a plastic container containing a saline solution up to 1 cm above the device. Pacing and defibrillation impedances were measured and were within the manufacturers’ required limits.

The CRT-D devices were programmed according to current clinical recommendations. The brady pacing mode remained *DDD* with a Lower Rate Limit of 70 min^−1^ and Rate Hysteresis *off*. The output paced the atrium and after 180 ms, it simultaneously paced both ventricles with a Pulse Amplitude of 2 V for the right atrium and ventricle, a Pulse Amplitude of 4 V for left ventricle and a Pulse Width of 0.5 ms. The LV pacing vector configuration was LV tip ->LV ring. Tachycardia detection zones were 160 min^−1^ for VT and 200 min^−1^. Shock therapy was on. All other parameters were set at nominal values as detailed in Table 1 and all therapies were on.

**Table 1:** Programming of CRT-D devices

Parameter	CRT-D setting
Pacing mode	DDD
Lower rate limit	70 bpm
Hysteresis	Off
Upper rate	Nominal 130 bpm
AV delay	Dynamic 120–180 ms
Ventricular pacing	BiV
RA, RV sense polarity	bipolar
LV pace polarity	bipolar LV tip -> LV ring
Rate smoothing	Off
VT zone	160 bpm
First VT therapy	Burst: 8 pulses at 81 % Scan: 8 pulses with 10 ms decr.
Second VT therapy	26 J
Third-last VT therapy	Maximum energy 41 J
VF zone	200 bpm
First VF therapy	26 J
Second-last VF therapy	Maximum energy 41 J

The type of programmer used was a Boston Scientific (formerly Guidant) Zoom Latitude model 3120 with the ability to wirelessly communicate (ZIP™ wandless telemetry) with a device. During the trial, the device was interrogated and a programmer screen was placed in front of the camera so that the traces and markers could be watched in the linear accelerator controller room. During radiation exposure, the strip from programmer was printed at a slow speed of 10 mm/s to document and check the pacing activity of the device.

An Elekta Precise linear accelerator was used to provide radiation with a photon beam of 6 MeV, 1a 0 × 10 cm field in aperture, distance to the source of 0.80 m and a dose rate of 800 cGy/min. This linear accelerator is used for all solid tumors. 6 MeV energy is dedicated mostly for areas cranially from the diaphragm. We measured 30 cm from the central beam on both sides, on the central beam and in the machine room.

## Results

During the study, we took seven measurements from different positions of the CRT-D can to the central beam of the linear accelerator using different radiation doses (Fig. 1). The CRT-D can with all three leads attached was placed in plastic containers filled with saline solution as was already described with the distance to radiation source of 0.80 m. The field in aperture was 10 × 10 cm in all seven cases. The temperature of the saline solution was measured before and after the irradiation of the system. The CRT-D markers and signals were watched using a camera focused on the programmer screen and also printed using an internal programmer printer. With this radiation dose, we did not notice any electromagnetic interference, any drop of biventricular pacing or any other contingencies.

### Case 1

The middle of the CRT-D can was placed 30 cm from the central beam on the patient deck in the direction from the machine room. The radiation dose was 10 Gy. The temperature of the saline solution remained unchanged at 16 °C.

### Case 2

The middle of the CRT-D can was placed 30 cm from the central beam on the patient deck in the direction to the machine room. The radiation dose was 10 Gy. The temperature of the saline solution however increased from 16.0 °C to 17.7 °C.

### Case 3

The middle of the CRT-D can was placed on the central beam on the patient deck. The radiation dose was 0.5 Gy. The temperature of the saline solution remained unchanged.

### Case 4

The middle of the CRT-D can was placed on the central beam on the patient deck. The radiation dose was 2 Gy. The temperature of the saline solution however decreased from 17.7 °C to 17.5 °C.

### Case 5

The middle of the CRT-D can was placed on the central beam on the patient deck. The radiation dose was 10 Gy. The temperature of the saline solution remained unchanged.

### Case 6

The middle of the CRT-D can was placed on the central beam on the patient deck. The radiation dose was increased 10 Gy. The temperature of the saline solution remained unchanged.

### Case 7

The CRT-D can with the lead system was placed in the machinery room of the linear accelerator. The radiation dose was 10 Gy. The temperature of the saline solution remained unchanged.

## Conclusions

After radiation exposure, the electrical parameters of the pacing system were checked with no significant differences compared to the initial values. Within the devices included in our measurement, there were no observed episodes of device reset, noise sensing, EMI or drop of biventricular pacing with irradiation up to a dose of 10 Gy. Compared to the typically-used maximum recommended dose of 2 Gy, the radiation doses used in our test were five times higher. For the CRT-D devices used in our study, there are no manufacturer’s recommendations or guidelines for managing patients undergoing radiotherapy. But with the knowledge that the market available cardiac implantable devices are made from similar components, we can generalize our results to similar CRT-Ds. We limited the results of this measurement to the use of a 6-MV beam with photons as a main component. At higher radiation levels, other effects must be taken into consideration.

Based on recommendations by Guidant from 2003 regarding scatter radiation and ICDs, ICDs may be five to ten times more sensitive to radiation damage than pacemakers since the operating instructions are stored in random access memory that may be more easily damaged by scatter radiation [21]. The best way to safety proceed with radiation therapy for patients with an implanted cardiac device would be simultaneous monitoring on the programmer for devices using wireless communication between the implant and the programmer.

## Discussion

The impact of therapeutic radiation on implanted devices is difficult to predict. Of course, the type of beam is an important consideration. High-energy photon beams have significantly more damaging effects than low-energy beams [22]. Besides this, multiple factors collectively determine the impact of radiation therapy on an implanted device. These factors include the type of implanted device, the distance from the implanted device to the radiation beam, the orientation of the beam to the implanted device, the dose rate and total dose delivered, shielding and the patient’s specific anatomy, physiology and heath condition [23]. Due to this variability, it is not possible to specify a safe radiation dosage or guarantee proper device functioning following exposure to ionizing radiation. The impact of ionizing radiation will also vary from one pulse generator to another and may range from no changes in function to a loss of pacing and defibrillation therapy. The effects of radiation exposure on an implanted device may remain undetected if patient follow-up is not performed after irradiation. For this reason, clinicians should monitor the device continuously or perform a follow-up right after the radiation session.

Unfortunately, the radiation sensitivity of cardiac implants also seems to be manufacturer dependent. In [10], eleven devices were directly irradiated by 6 mV beams, each to a cumulative dose of 20 Gy. Four of the 11 devices experienced a complete loss of function after only 1.5 Gy of radiation. In our study, we did not see any negative influences up to several tens of Gy. Comparing our results with other older studies, the maximum destructive single dose might vary in a staggering range from 1 up to 80 Gy. It is theoretically possible to assess the risk of hardware circuitry damage. However, random alteration of device memory or electrical components by scatter particles is an issue and it is difficult to predict the safe level. Some devices may be susceptible to other sources of radiation. For example, thermal neutrons can be generated by linear accelerators and they can adversely affect device behavior [24, 25].

As it is not possible to absolutely shield the devices, implanted systems will always be somewhat susceptible to the effects of radiation, regardless of any precautions taken. Because most of the implanted devices now are wireless telemetry enabled, our recommendation is to continuously wirelessly monitor the device during radiotherapy sessions.
